# Prior information-based subtraction radiography for enhanced tumor visibility in image-guided radiotherapy

**DOI:** 10.3389/fonc.2026.1750996

**Published:** 2026-03-18

**Authors:** Yanle Hu, Shuai Leng

**Affiliations:** 1 Department of Radiation Oncology, Mayo Clinic in Arizona, Phoenix, AZ, United States; 2 Department of Radiology, Mayo Clinic, Rochester, MN, United States

**Keywords:** 2D X-ray, on-board imaging, radiotherapy, subtraction radiography, tumor visualization

## Abstract

**Purpose:**

Two-dimensional (2D) X-ray imaging is routinely used in radiotherapy for patient alignment but offers limited tumor visibility due to anatomical overlap. We propose a novel technique, prior information-based subtraction (PIBS) radiography, to enhance tumor visualization on 2D X-ray and facilitate more accurate patient alignment.

**Materials and methods:**

PIBS X-ray improves tumor visualization on 2D X-ray by subtracting signal contributions from non-essential (NE) tissues, which can be obtained from the planning CT (prior information). To demonstrate the feasibility, we acquired four CT scans using a thorax phantom with a spherical lung target, representing the planning CT and three treatment-day CTs capturing the patient’s positions with the lung tumor at three different locations, respectively. NE tissues, defined as structures outside the thoracic cavity, were segmented from the planning CT. On treatment days, we aligned NE tissues based on bony structures via 3D/3D or 2D/2D registration and applied the calculated shifts so the patient’s position matched that in the planning CT. To simulate 2D X-rays, digitally reconstructed radiographs (DRRs) were generated from the planning CT of NE tissues and aligned with treatment-day CTs. Subtracting NE tissue contributions from conventional 2D X-ray yielded PIBS X-ray. PIBS X-ray images were compared with conventional X-ray images to assess tumor visibility.

**Results:**

PIBS X-ray provided better tumor visualization across all projection angles and all tumor locations compared to conventional X-ray, even for certain cases where the tumor was completely indistinguishable on conventional X-ray. Both 3D/3D and 2D/2D registrations produced acceptable alignment of NE tissues because of the clear visibility of bony structures.

**Conclusion:**

PIBS X-ray is a promising technique to enhance tumor visualization on 2D X-ray. In this study, we demonstrated its potential in improving radiotherapy on-board imaging using a thorax phantom. In the future, we will explore strategies for integrating this technology into clinical workflows and evaluate its clinical benefits in radiotherapy patients.

## Introduction

Radiotherapy utilizes on-board imaging (1–7) to position patients in alignment with their planning computed tomography (CT) setups. This process ensures that tumors are accurately positioned within the pre-optimized radiation fields for daily treatments. It is an essential component in radiotherapy treatment of cancers, aiming at delivering the prescribed radiation dose to the tumor while minimizing radiation doses to surrounding organs-at-risk (OARs). The ability to visualize tumors using on-board imaging directly impacts patient setup and, thus, dose delivery accuracy.

Currently, most radiotherapy on-board imaging systems are based on X-ray imaging (2–6). However, X-ray imaging has significant limitations in visualizing the tumor due to low soft tissue contrast. This is particularly true for 2D X-rays (2, 5) because of anatomy overlapping, in which all tissues along the X-ray paths contribute to the final image. While 3D X-ray imaging such as cone-beam CT (CBCT) provides better visualization of internal anatomy (4, 6), they come with higher imaging doses (8) and slower acquisition speed (9), making them unsuitable for real-time tracking of tumor motion. Visualizing tumors directly on 2D X-ray offers many clinical benefits. It has less imaging dose and is faster to acquire. More importantly, it allows direct tracking of tumor motion, instead of surrogate motion, through continuous 2D X-ray imaging.

Enhancing tumor visibility on 2D X-ray imaging has attracted significant interest in radiotherapy. Current developmental efforts primarily focus on two approaches. One is based on dual-energy X-ray imaging (10–12), leveraging differential changes in attenuation coefficients for bones and soft tissues between two X-ray energies to suppress signal contributions from bones. This approach could enhance tumor visibility when the tumor overlaps with bony structures. However, tumor contrast enhancement is limited as the remaining soft tissues (e.g., muscle) still contribute to 2D X-ray imaging, which reduces tumor contrast. The other approach is based on artificial intelligence (AI) (13, 14), which uses trained models to predict tumor shape and location from conventional X-ray images. While promising, this approach lacks an effective way to verify the prediction accuracy for individual patients, as tumors are difficult to visualize on 2D X-ray.

To address these challenges, we propose a novel approach, termed prior information-based subtraction (PIBS) radiography, to enhance tumor visualization on 2D X-ray, leveraging prior knowledge about patient anatomy available in the planning CT. PIBS X-ray is based on the rationale that tumors can be better visualized on 2D X-ray by removing signal contributions from certain tissues that remain relatively stable during the radiotherapy treatment course. These tissues, termed non-essential (NE) tissues, can be identified from the planning CT (15) and treated as known prior information on treatment days. For example, tissues outside the thoracic cavity can be deemed as NE tissues for lung cancer. Based on NE tissues obtained from the planning CT, it is possible to simulate the contributions of NE tissues to 2D X-ray and subtract those from the treatment-day 2D X-ray to enhance tumor visualization. Compared to the dual-energy method, PIBS X-ray has better tumor enhancement as it removes signal contributions from both bones and soft tissues (i.e., NE tissues). In contrast to the AI approach, PIBS X-ray improves tumor visibility purely through imaging and does not have uncertainties associated with model prediction. The primary goal of this work is to introduce the methodology and demonstrate the feasibility of PIBS X-ray imaging using a thorax phantom.

## Materials and methods

### Overview of PIBS X-ray imaging

To explain how the PIBS X-ray works, we used lung cancer as an example. Lung cancer was selected because of two considerations. Firstly, identifying NE tissues is straightforward. All tissues outside the thoracic cavity (e.g., muscles, bones) remain stable during radiotherapy treatment and can be deemed as NE tissues. Secondly, solid lung tumors maintain a good contrast relative to the lung tissue. Once signal contributions from NE tissues are removed, lung tumors become clearly visible on 2D X-ray as the lung tissue has a much lower density compared to solid lung tumors.

**Figure 1:**
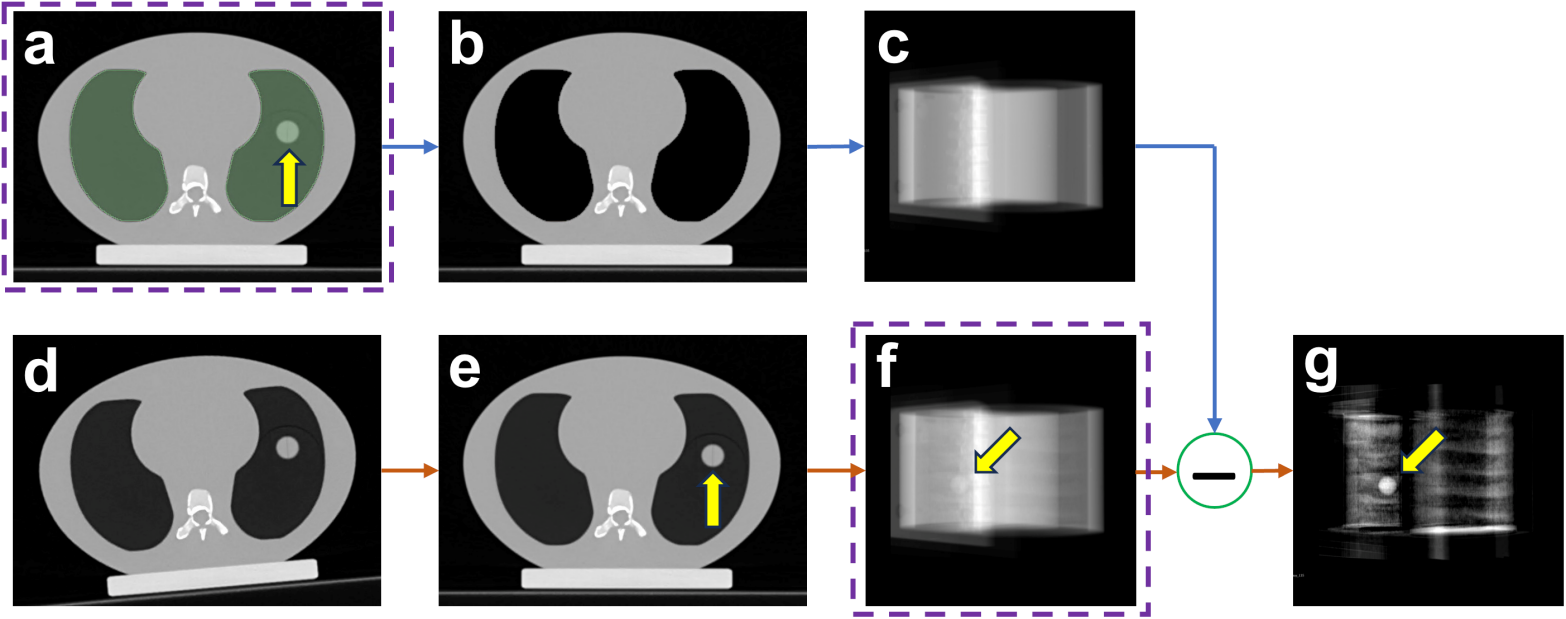
PIBS X-ray imaging workflow. **(A)** The planning CT, **(B)** the planning CT of NE tissues, **(C)** simulated X-ray of NE tissues, **(D)** treatment-day initial patient position on couch as represented by treatment-day CT, **(E)** treatment-day patient position after aligning NE tissues as represented by shifted treatment-day CT, **(F)** simulated treatment-day X-ray, and **(G)** PIBS X-ray created by subtracting the simulated X-ray of NE tissue **(C)** from treatment-day X-ray **(F)**. Comparing **(G)** with **(F)**, lung tumor visualization is significantly improved on PIBS X-ray. Note: The planning CT **(A)** and treatment day X-ray after couch shift **(F)**, when aligned based on bony structures, are already part of the patient’s medical record through the standard-of-care practice.

**Figure 1** illustrates the PIBS X-ray imaging workflow. Radiotherapy typically starts with a CT scan acquired while the patient is positioned on the CT couch. This CT scan, referred to as the planning CT (**Figure 1A**), serves as the anatomical reference for radiotherapy treatment planning. The planning CT provides detailed anatomical information about patients, which can also be used as prior information on treatment days. From the planning CT, NE tissues, i.e., tissues outside the thoracic cavity, can be obtained by segmenting the entire lung (including lung tumors) and overriding its CT numbers to that of the air, as shown in **Figure 1B**. Based on the planning CT of NE tissues, we can simulate 2D X-rays of NE tissues (**Figure 1C**) using geometry setups that match those in the treatment room. On treatment days, the patient is initially positioned based on in-room alignment lasers (**Figure 1D**). Either 3D CBCT or 2D X-ray images are performed to capture the patient’s initial position on the treatment couch. A 3D/3D co-registration based on CBCT, or 3D/2D, or 2D/2D co-registration based on 2D X-ray is performed, utilizing structures with high contrast (e.g., bones, diaphragm), to calculate couch shifts to align NE tissues. After applying these couch shifts, the patient’s NE tissues on treatment days match those in the initial planning CT (**Figure 1E**). At this position, a set of X-ray images is acquired (**Figure 1F**). Given that NE tissues are aligned, subtracting the simulated 2D X-ray of NE tissues (**Figure 1C**) from the treatment-day 2D X-ray (**Figure 1F**) yields the PIBS X-ray with enhanced tumor contrast (**Figure 1G**).

### Phantom

We used the CIRS Dynamic Thorax Phantom (Model 008A) to demonstrate the feasibility of PIBS X-ray. The phantom includes an anthropomorphic body mimicking an average human thorax and a lung equivalent rod containing a spherical target mimicking a lung tumor. The phantom is controlled by CIRS Motion Control Software and can produce independent 1D surrogate and 3D target motion.

### Image acquisition

The CIRS Dynamic Thorax Phantom was scanned using a Siemens CT scanner (Somatom AS-64, Siemens Healthineers, Erlangen, Germany). The phantom was first placed on the CT couch with the assistance of external lasers. The spherical tumor was set at the most inferior position, simulating the end of inhalation. A 3D volumetric CT image set was acquired using a standard thorax protocol. Key imaging parameters included 120 kVp, automatic tube current modulation (CARE Dose4D, Siemens Healthineers) with a reference mAs of 280, 1.0 s gantry rotation, 0.8 pitch, 2 mm slice thickness, 50 cm reconstructed field of view (FOV), and SAFIRE iterative reconstruction with a strength of 3. This CT image set corresponded to the planning CT acquired at the beginning of radiotherapy.

After the first CT scan, the phantom was re-positioned, adding shifts in six dimensions (three translational and three rotational shifts), to simulate the patient’s initial setup position on treatment days. Using the same image acquisition parameters described in the previous paragraph, three volumetric CTs were acquired, with the tumor being at the end-of-inhalation, mid-inhalation, and end-of-exhalation positions, respectively. To facilitate the description of the PIBS X-ray approach, these CT image sets are referred to as the treatment-day CT. Given that respiration-induced motion is often in the superior/inferior direction, in this work, we only introduced motion in this direction to demonstrate feasibility.

### Simulation software

We used the 3D Slicer (16) software (version 5.8.0, https://www.slicer.org/) to simulate PIBS X-ray and demonstrate its feasibility. In this study, we used the Segment Editor module for contouring and CT number overriding, the SlicerElastix module for 3D image co-registration, the Resample Scalar/Vector/DWI Volume module for resampling the co-registered images into the normal axes (i.e., axial, sagittal and coronal), the Plastimatch (17) module for generating digitally reconstructed radiograph (DRR) (18, 19), and the Subtract Scalar Volumes module for creating PIBS X-ray by subtracting 2D X-ray of NE tissues from treatment-day X-ray.

### Alignment of NE tissues on treatment days

For PIBS X-ray to work, NE tissues need to remain stable throughout the treatment course and can be reliably aligned between the day of acquiring the planning CT and treatment days using existing on-board imaging capabilities (3D CBCT or 2D X-ray). In lung cases, alignment of NE tissues can be achieved based on bony structures such as vertebral bodies within NE tissues. These structures have good contrast and are clearly visible on both 3D CBCT and 2D X-ray. Their position remains stable relative to other NE tissues. Therefore, they serve well for the alignment purpose.

In this study, we used the treatment-day CT to simulate the patient’s initial position on the couch on a treatment day. This position was set to be different from that in the planning CT to mimic the real case scenario. Clinically, depending on the available on-board imaging capabilities, either 3D CBCT or two orthogonal 2D X-ray images can be acquired to align NE tissues. By co-registering on-board images to the planning CT based on bony structures, couch shifts can be obtained and applied to bring the patient’s treatment-day couch position into alignment with that in the planning CT.

To simulate 3D/3D co-registration, we used the treatment-day CT as a substitute for CBCT and co-registered it to the planning CT using the SlicerElastix module. The calculated shifts were applied to the treatment-day CT and resampled to the normal axis to simulate the process of couch shifting. The resampled (or shifted) treatment-day CT represented the patient’s position after couch shift with NE tissues being aligned.

To simulate 2D/2D co-registration, we used DRRs generated from the treatment-day CT as a substitute for 2D X-ray images and co-registered them with DRRs generated from the planning CT. DRR represents the primary signal of 2D X-ray, which is sufficient for demonstrating the feasibility of PIBS X-ray. DRRs at projection angles of 45° and 135° were created, which represented a common setup for orthogonal X-ray. Key parameters included 100 cm SAD (source-to-axis distance) and 150 cm SID (source-to-imager distance), 1,024 × 1,024 resolution, and 0.0417 cm × 0.0417 cm spacing. Exponential mapping and intensity inversion were applied to match the appearance of a routine X-ray. Manual co-registration was performed between DRRs of the treatment-day CT and those of the planning CT based on bony structures. The obtained couch shifts were applied to the treatment-day CT. The shifted treatment-day CT was resampled to the normal axis, reflecting the patient’s position after couch shift with NE tissues being aligned.

### Planning CT of NE tissues

In 3D Slicer, we used the Segment Editor to contour the entire lung (including the lung tumor) on the planning CT and then override its CT number to −1,024 HU, corresponding to the lowest CT number in the 12-bit CT scanner. This operation was equivalent to replacing the lung tissue with air, as any CT numbers lower than −1,000 HU map to an X-ray attenuation coefficient of 0. As a result, only tissues outside the thoracic cavity (i.e., NE tissues for lung cancer) remained on the output images. These output images were referred to as the planning CT of NE tissues.

### PIBS X-ray

**Figure 2:**
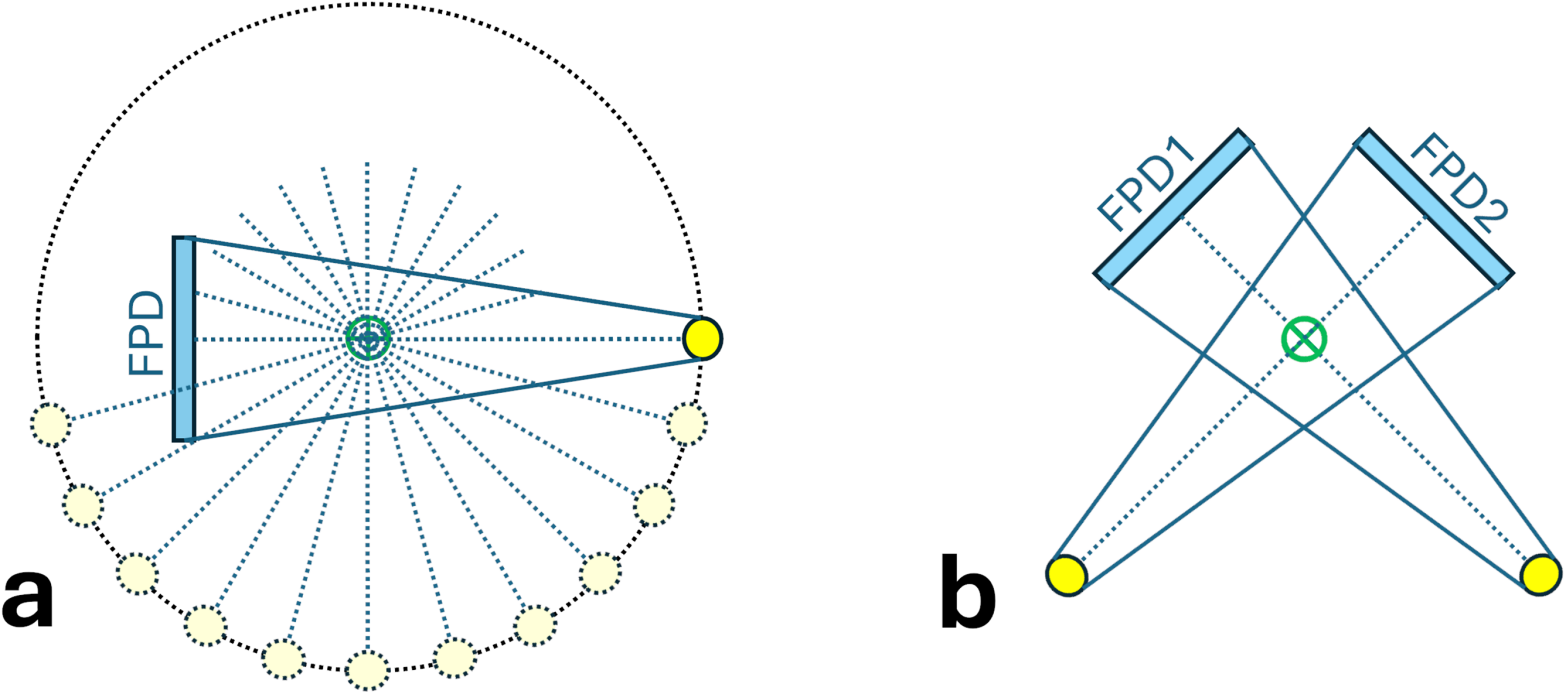
Illustration of projection angles used to generate conventional and PIBS X-ray images for comparison. **(A)** For on-board imaging systems with 3D CBCT capabilities, projection angles from 0° to 179° at an interval of 15° were selected. **(B)** For on-board imaging systems with only 2D X-ray capabilities, projection angles of 45° and 135° were selected. These provide a good sampling of possible clinical scenarios.

After NE tissues were aligned, PIBS X-rays were generated by directly subtracting simulated X-rays (i.e., DRRs) of NE tissues from simulated X-rays of the shifted treatment-day CT. PIBS X-rays were compared to simulated X-rays of the shifted treatment-day CT (i.e., conventional X-ray) for evaluating their potential in enhancing lung tumor visualization. For radiotherapy systems equipped with CBCT, the on-board imaging unit is usually mounted on the treatment gantry and thus can rotate together with the gantry. Therefore, 2D X-ray images can be taken at any gantry angles. In such cases, simulated X-ray images were created at projection angles from 0° to 179° at an interval of 15° (**Figure 2A**), providing a complete sampling of the entire clinical scenarios. Projection angles from 180° to 359° are opposite to those from 0° to 179°. These offer redundant information and, therefore, were excluded from this study. For radiotherapy systems with only the 2D X-ray capability, a common setup includes two orthogonal X-ray systems at the angles of 45° and 135° (**Figure 2B**). For this scenario, only those two angles were used to generate simulated PIBS X-ray and conventional X-ray images for evaluation.

### PIBS X-ray for motion tracking

The potential of PIBS X-ray for motion tracking was demonstrated using the planning CT and three treatment-day CTs with the lung tumor being at the end-of-inhalation, mid-inhalation, and end-of-exhalation positions, respectively. Since NE tissues remained relatively stable while the lung tumor moved to different positions, image co-registration was performed between one treatment-day CT (e.g., tumor at the end-of-inhalation position) and the planning CT. The transformation matrix obtained was applied to all three treatment-day CTs to mimic couch shifting. For demonstrating feasibility, the 3D/3D co-registration and the projection angle of 150° were used as an example for creating PIBS X-ray because it represented the most challenging case. PIBS X-rays were created and compared to the simulated conventional X-rays for the three tumor positions.

## Results

**Figure 3:**
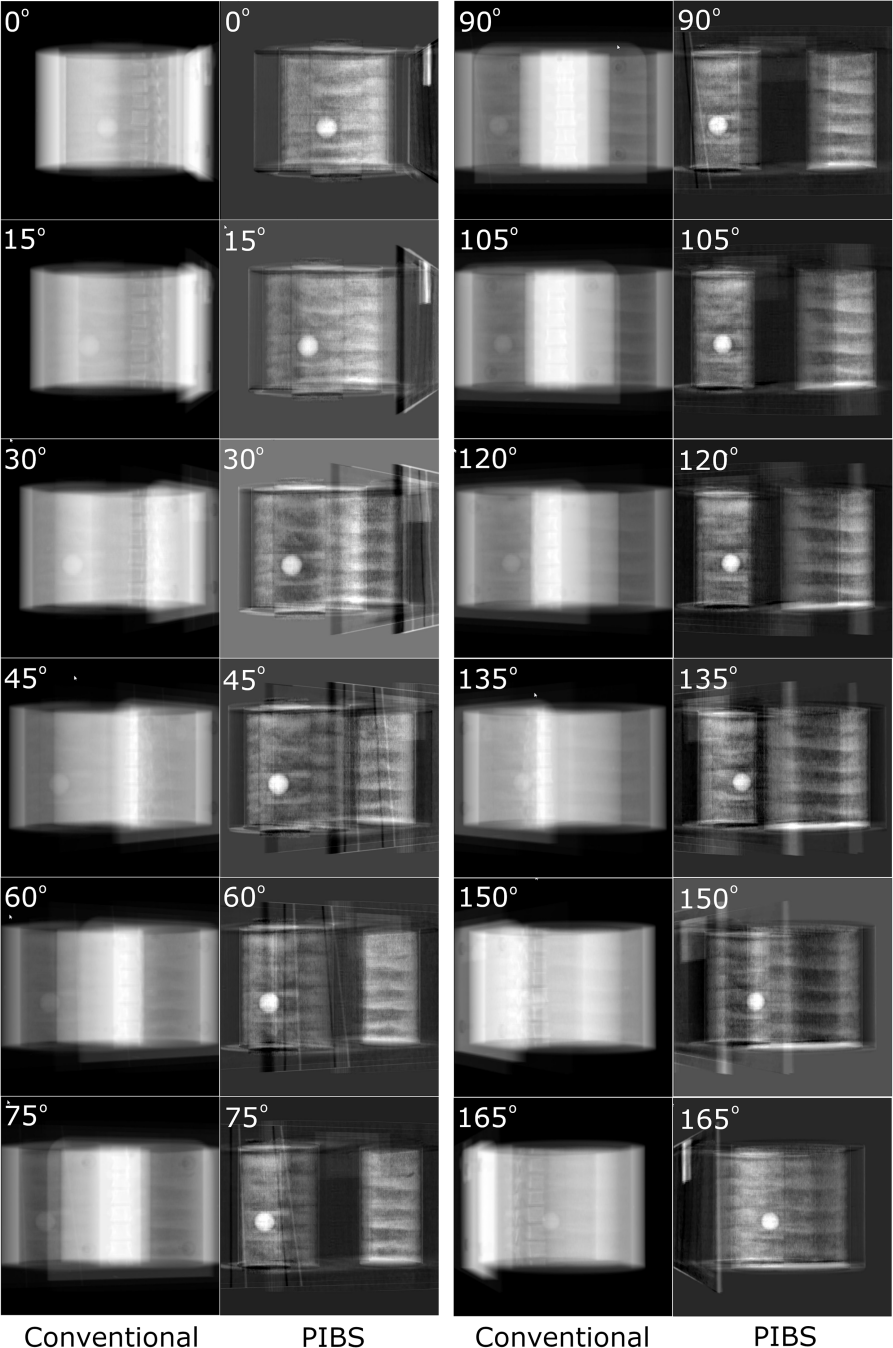
Comparison of conventional and PIBS X-ray images at projection angles of 0° to 179° at an interval of 15° for on-board imaging systems with 3D CBCT capabilities. Automatic window/level settings selected by 3D Slicer were used to display those images. PIBS X-ray was able to remove signal contributions from NE tissues and significantly enhance lung tumor visualization.

**Figure 4:**
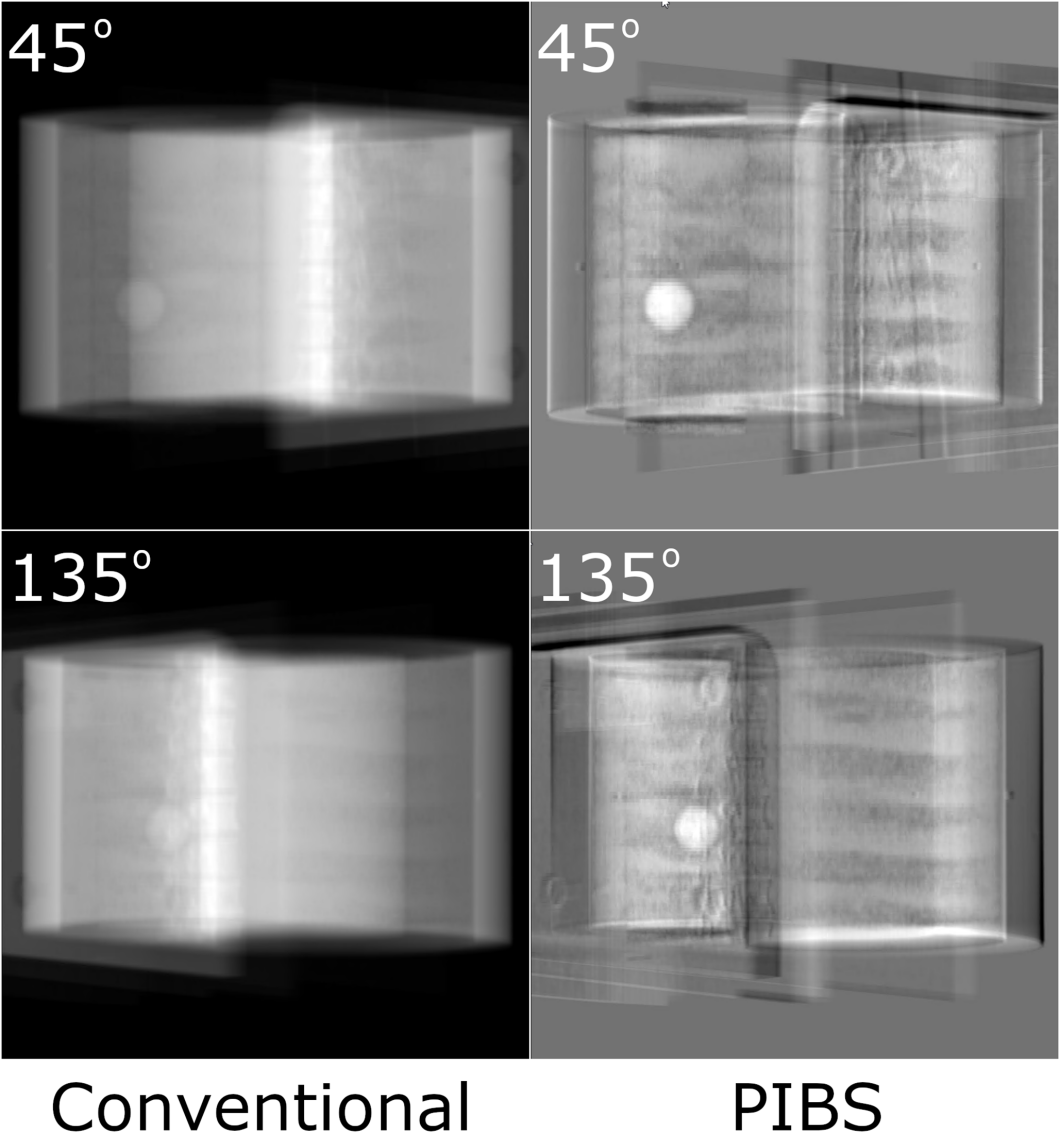
Comparison of conventional and PIBS X-ray images at projection angles of 45° to 135° for on-board imaging systems with only 2D X-ray capabilities, representing the most common setup for these systems. Automatic window/level settings selected by 3D Slicer were used to display those images. Enhancement in tumor visualization is clear on PIBS X-ray.

PIBS X-rays were generated based on both 3D/3D registration to simulate systems with the 3D CBCT capability and 2D/2D registration to simulate systems with only the 2D X-ray capability. These PIBS X-ray images were compared to the conventional X-ray images to assess their capabilities in enhancing lung tumor visualization. **Figure 3** shows the results based on 3D/3D registration. Conventional X-ray and PIBS X-ray images from 12 projection angles were displayed using the automatic display window/level selected by 3D Slicer. Specifically, the window/level setting was 63,713/−33,679, 65,326/−32,872, 63,537/−33,767, 63,412/−33,829, 64,420/−33,325, 63,731/−33,670, 63,636/−33,717, 63,036/−34,017, 64,322/−33,374, 63,866/−33,602, 64,268/−33,401, and 65,302/−322,84 for conventional X-ray, respectively, and 10,933/3,172, 11,125/2,326, 9,909/288, 8,418/2,081, 9,611/3,032, 10,438/3,861, 10,145/3,819, 10,180/3,984, 9,112/3,644, 8,181/2,739, 11,987/2,119, and 11,413/3,901 for PIBS X-ray, respectively. The enhancement in lung tumor visualization is clearly visible. Given that the phantom is composed of uniform materials except for vertebral bodies, the lung tumor is visible even on conventional X-rays for some projection angles where the lung tumor is in locations with a relatively uniform background (e.g., projection angle of 105°). However, for certain projection angles (e.g., 150°), it is completely masked by vertebral bodies and becomes indistinguishable. In contrast, the lung tumor stands out in PIBS X-rays. It is clearly visible for all projection angles. The feasibility of PIBS X-ray for systems only equipped with the 2D X-ray capability is demonstrated in **Figure 4**, in which the alignment of NE tissues was based on 2D/2D registration. Again, the automatic display window/level selected by 3D Slicer was used in displaying the conventional and PIBS X-ray images. Specifically, the window/level setting for 45° and 135° projection angles was 63,496/−33,787 and 64,525/−33273 for conventional X-ray, respectively, and 13,065/−134 and 14,804/762 for PIBS X-ray, respectively. The projection angles of 45° and 135° were selected as they represent the most common setup. Even with 2D/2D registration, we could also achieve good results. In both scenarios, PIBS X-ray was able to significantly improve lung tumor visualization, compared to conventional X-ray, demonstrating its clinical potential.

**Figure 5:**
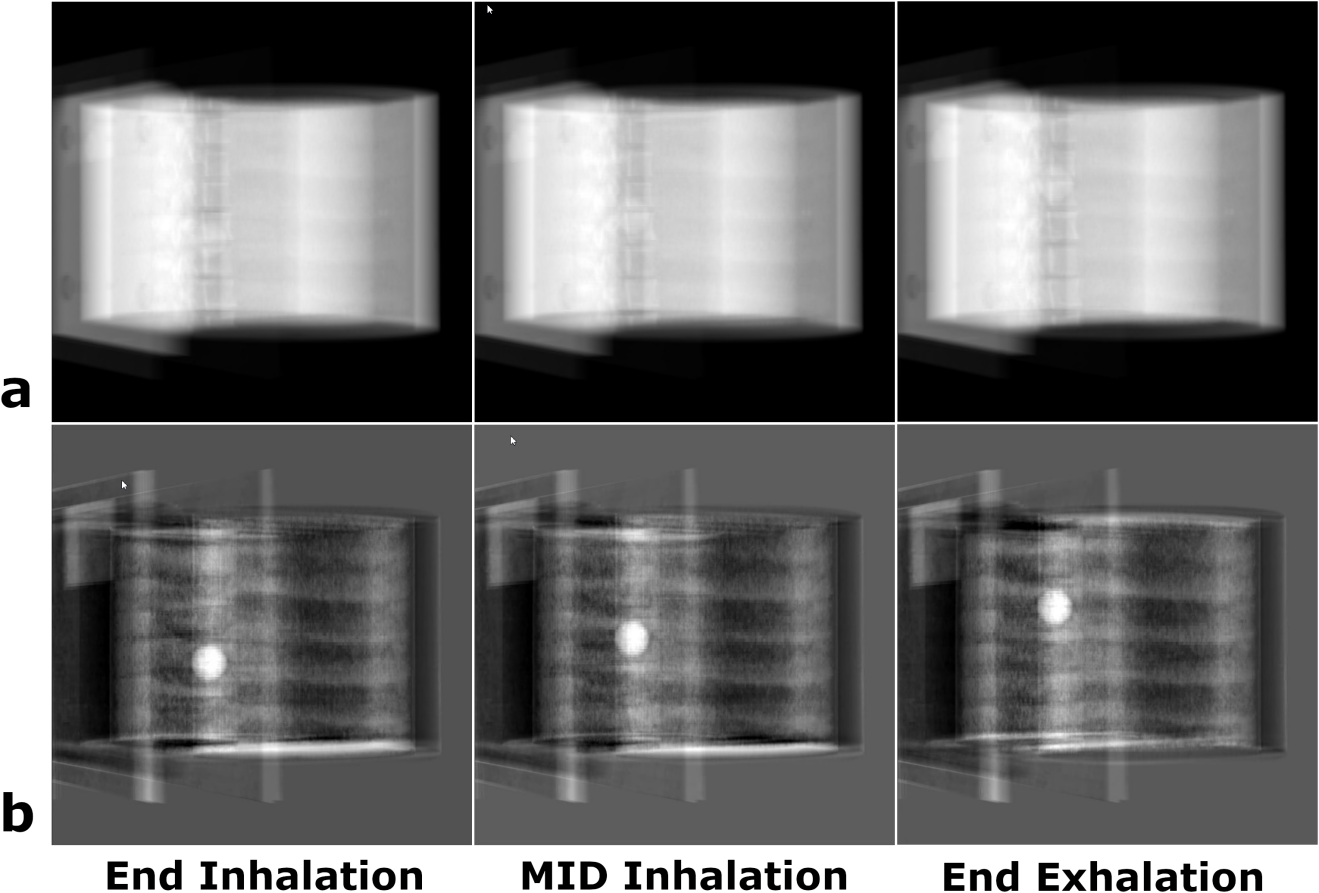
Demonstration of the feasibility of lung tumor motion tracking using PIBS X-ray. Three treatment-day CTs with the lung tumor at different locations were used to simulate respiration-induced tumor motion. Conventional **(A)** and PIBS **(B)** X-ray images were generated at the projection angle of 150° based on 3D/3D registration, as it represented the most challenging case visualizing the lung tumor. Automatic window/level settings from 3D Slicer were used to display the images. The lung tumor had a distinct contrast on PIBS X-ray but was barely visible on conventional X-ray, showing the potential of PIBS X-ray in lung tumor motion tracking.

**Figure 5** demonstrates the feasibility of using PIBS X-ray to achieve lung tumor motion tracking. Given that NE tissues are less subject to respiratory motion, they can be obtained from the planning CT regardless of the lung tumor location. In this case, we used the planning CT at end-inhalation to obtain NE tissue information. DRRs of NE tissues were created from the planning CT of NE tissue at end-inhalation (or any respiratory state). They were subtracted from DRRs of the treatment-day CTs at end-inhalation, mid-inhalation, and end-exhalation to generate PIBS X-rays at three tumor locations. Results were compared to conventional X-rays. At the projection angle of 150°, the lung tumor was barely visible on conventional X-rays but had very distinct contrast on PIBS X-rays (the window/level setting for the end-inhalation, mid-inhalation, and end-exhalation was 64,248/−33,401, 63,591/−33,740, and 63,966/−33,552 for conventional X-ray, respectively, and 11,987/2,119, 12,088/1,612, and 11,842/1,774 for PIBS X-ray, respectively).

## Discussion

Radiotherapy treatment of lung cancer is always complicated by respiratory motion (20, 21). Current on-board imaging systems have very limited capabilities in providing accurate tumor location information. 3D CBCT provides good volumetric images for positioning patients who have static tumors such as brain, head and neck, and pelvic cancers. However, its slow acquisition speed (ranging from 6 to 60 s) makes it insufficient to track respiration-induced tumor motion and therefore cannot support motion management strategies such as gated treatment. In these situations, 2D X-ray imaging (i.e., fluoroscopy) provides a viable option from the perspective of temporal resolution. Unfortunately, conventional 2D X-ray suffers from low tumor tissue contrast due to the anatomy overlap along the projection path. As a result, tumors are often not visible, making real-time tumor motion tracking unreliable or impossible. Clinically, we rely on the external surrogate to determine tumor position. However, it is well known that the external surrogates and internal tumor locations do not always correlate well (22–24). To address this challenge, several approaches have been proposed, but their effectiveness has been limited. For example, dual-energy X-ray imaging exploits differential attenuation changes for soft tissue and bone between different energy levels to suppress bony structures. However, this does not sufficiently enhance tumor visibility because soft tissues still obscure the tumor. More recently, AI-based methods have been used to predict tumor position based on conventional 2D X-ray, but these rely on model-based inference rather than direct visualization, and their accuracy cannot be verified for individual patients.

The proposed PIBS X-ray technique addresses these challenges by using prior information to remove signal contributions of NE tissues from conventional X-ray, including both bony structures and soft tissues that remain stable during treatment. This is a purely imaging-based approach, and its accuracy can be directly verified from the images themselves. As a result, PIBS X-ray provides a practical and effective way of visualizing and tracking real-time tumor motion, enabling motion management strategies based on tumor position, instead of surrogate position. PIBS X-ray does require that the tumor still remains visible when NE tissues are removed. Lung cancer is therefore an excellent application, as lung tumors typically exhibit strong contrast against lung tissue. For abdominal cancers, however, determining which tissues qualify as NE tissues is more complex because tumors are often surrounded by soft tissues with similar attenuation characteristics. Adopting PIBS X-ray for abdominal cancers requires further consideration to determine its feasibility.

There are several potential clinical scenarios that could benefit from PIBS X-ray. First, it provides accurate lung tumor location information that can be used to accurately position patients on the treatment couch so the tumor aligns with its planned location and remains within the PTV. This is particularly valuable when the motion is large. Direct visualization of the tumor position would ensure more reliable patient setup and ultimately improve treatment accuracy. With further development of this technology, it could enable real-time lung tumor motion tracking, allowing us to implement motion management strategies based on actual tumor position rather than surrogate position. It has great potential in improving tumor dose coverage while minimizing OAR doses.

PIBS X-ray is based on a subtraction operation. Therefore, its performance depends on the accurate alignment of NE tissues. Being able to visualize NE tissues on images (either 3D CBCT or 2D X-ray) acquired from the on-board imaging unit plays an essential role in creating high-quality PIBS X-ray images. Clinically, NE tissues contain bony structures that can be clearly visualized on both 3D CBCT and conventional 2D X-ray. Aligning based on bony structures is practically achievable. In fact, this is routinely used in clinical practice. Given that non-bony NE tissues (e.g., muscle) maintain a stable position relative to bony NE tissues, the entire NE tissues are in alignment with those in the planning CT once bony structures are aligned, making it ready for the subtraction operation. Being able to align NE tissues enables us to use the planning CT of NE tissues to simulate X-rays of NE tissues. It mitigates time constraints in generating the simulated X-rays of NE tissues, as there is usually sufficient time (e.g., days) between the acquisition of the planning CT and the delivery of radiotherapy treatments. These X-rays of NE tissues can be precalculated and ready for use on treatment days. We acknowledge that image registration may not be perfect, and there exists registration uncertainty. Imperfect registration may lead to increased background signals, reducing our ability to visualize the lung tumor. However, the central location of the tumor will remain the same, which is more important in determining the respiration-induced tumor location change.

In this study, we used simulated superior/inferior motion to demonstrate the feasibility of PIBS X-ray for tracking lung tumor motion. Although this simplified motion pattern may not fully capture the complexity of real patient tumor motion, the prior information used in PIBS X-ray only involves NE tissues, which are outside the thoracic cavity. Tumor motion, whether 1D or 3D, does not affect the subtraction operation itself. Therefore, for the purpose of demonstrating feasibility, this simplification would not change the results and conclusion presented here. More comprehensive evaluation based on real patients will be conducted in future studies.

PIBS X-ray uses conventional X-ray and planning CT images acquired through the current standard-of-care practice. It does not require additional image acquisition and therefore will not add imaging dose to patients. The tumor location information provided by PIBS X-ray is completely in addition to what is currently available in routine clinical practice. We do not remove conventional X-rays, and these images can also be used in clinical practice to provide standard-of-care treatments or whenever deemed appropriate. PIBS X-ray offers new information utilizing existing on-board imaging hardware, which can be used in combination with conventional X-ray, to facilitate treating physicians making more informed clinical decisions and bringing better radiotherapy treatments to lung cancer patients.

PIBS X-ray has a natural fit for lung cancer. However, it may have potential applications for other disease sites as well. For example, prostate cancer patients receiving radiotherapy may have fiducial markers placed inside the prostate to assist patient alignment. To minimize dose disturbance, markers made of low Z materials (e.g., carbon-coated zirconium oxide) are preferred over those made of high Z materials (e.g., gold). For radiotherapy systems equipped with only 2D X-ray imaging capabilities (e.g., some proton systems), visualizing carbon fiducial markers on 2D X-ray for obese patients can become challenging, especially for certain treatment angles, due to excessive tissue overlapping in the pelvic region. Utilizing PIBS X-ray, it is possible to eliminate signal contributions from the pelvic bone as well as surrounding muscle and fat tissues to enhance visualization of fiducial markers on 2D X-ray.

One limitation of the current study is that the advantages of PIBS X-ray were demonstrated through qualitative evaluation. Although quantitative evaluation could be more objective and thus preferred, it is very challenging to identify suitable quantitative metrics for evaluation. For example, when the lung tumor overlaps with vertebral bodies (**Figure 3**, projection angle 150°), where to place the analysis regions of interest (ROIs) can be very subjective and affect the calculated contrast-to-noise ratio (CNR) dramatically. Another limitation of the study is that the feasibility of PIBS X-ray was demonstrated using a phantom study. In clinical settings, patient studies may present additional complexities: for example, NE tissues may not remain completely rigid, real patients have more heterogeneity, and patient anatomy may change during the course of treatment. This can cause challenges in NE tissue alignment, reduce the effectiveness of the subtraction operation, and increase background signals. Dramatic background signal increases may occur near the boundaries of the lung tissue due to imperfect registration. However, these are expected to be distant from the tumor and thus may not affect tumor visualization. In addition, we can project the PTV contour to PIBS X-ray images to provide a coarse estimate of tumor location that will help separate the tumor from residual artifacts as well. It is reasonable to expect that PIBS X-ray will improve tumor visibility for many patients compared to conventional X-ray. In scenarios where PIBS X-ray does not provide help due to excessive artifacts, or whenever there are any doubts, we could still proceed with conventional X-ray. So, using PIBS X-ray in addition to conventional X-ray will either improve or at least maintain the existing imaging capabilities.

## Conclusion

In this work, we introduced a novel methodology to enhance tumor visualization in 2D X-ray images for radiotherapy on-board imaging and demonstrated its feasibility using a phantom study. The proposed approach shows strong potential for the radiotherapy treatment of lung cancer by enabling better patient alignment and facilitating real-time lung tumor motion tracking to optimize radiotherapy treatment. Translating the potential of PIBS X-ray into improved patient care will require further investigation on integrating it into clinical workflow and validating its performance through patient studies.

## Data Availability

The raw data supporting the conclusions of this article will be made available by the authors, without undue reservation.
